# Quantitative Parameters of the Body Composition Influencing Host Seeking Behavior of *Ixodes ricinus* Adults

**DOI:** 10.3390/pathogens10060706

**Published:** 2021-06-05

**Authors:** Joanna Kulisz, Katarzyna Bartosik, Zbigniew Zając, Aneta Woźniak, Szymon Kolasa

**Affiliations:** 1 Chair and Department of Biology and Parasitology, Faculty of Health Sciences, Medical University of Lublin, Radziwiłłowska 11 St., 20-080 Lublin, Poland; katarzyna.bartosik@umlub.pl (K.B.); zbigniew.zajac@umlub.pl (Z.Z.); aneta.wozniak@umlub.pl (A.W.); 2 Polesie National Park, Lubelska 3a St., 22-234 Urszulin, Poland; szymon.kolasa@poleskipn.pl

**Keywords:** *Ixodes ricinus*, tick parameters, questing ticks, host-seeking behavior

## Abstract

*Ixodes ricinus*, a hematophagous arthropod species with great medical importance in the northern hemisphere, is characterized by an ability to survive prolonged periods of starvation, a wide host spectrum, and high vector competence. The aim of the present study was to determine the quantitative parameters of questing *I. ricinus* ticks collected in eastern Poland during the spring peak of their activity. The study consisted in the determination of quantitative parameters characterizing *I. ricinus* females and males, i.e., fresh body mass, reduced body mass, lipid-free body mass, water mass, and lipid mass and calculation of the lipid index. A statistically significant difference was observed between the mean values of the lipid index in females collected during the first and last ten days of May, which indicates the progressive utilization of reserve materials in the activity period. Higher activity of *I. ricinus* female ticks was observed during the last ten days of May despite the less favorable weather conditions, indicating their strong determination in host-seeking behaviors accompanying a decline in the lipid content and the use of the “now or never” strategy.

## 1. Introduction

Ticks (Acari: Ixodida) are the most important vectors of pathogens among arthropods occurring in the Western Palearctic [1,2]. Species representing the *Ixodes ricinus* complex have the greatest medical importance associated with their ability to transmit pathogens to humans and their role in the maintenance and circulation of tick-borne pathogens and diseases in animals [3,4,5,6].

In Poland, *Ixodes ricinus* ticks have the greatest medical importance [7,8,9,10,11,12]. The species has been shown to transmit numerous harmful microorganisms, including spirochetes, rickettsiae, protozoa, and viruses [9,13,14]. The genetic material of many other microorganisms has been detected in *I. ricinus* ticks as well. However, their pathogenicity to humans is still being investigated [9,15,16]. The most common direct effects of *I. ricinus* infestations in humans are skin lesions, i.e., redness at the bite site [7,17,18]. Additionally, the components of tick saliva can cause local and systemic allergic reactions. The literature has also reported cases of allergy to red meat induced by IgE Ab against alpha-gal in patients infested by this tick species [19,20,21,22].

The ability of ticks to transmit pathogens is influenced by external factors regulating their host-seeking activity in the environment and modifying the risk of attacks (i.e., temperature, relative humidity, saturation deficit, photoperiod, presence of potential hosts) [23,24,25], and internal factors regulated at the level of genes and gene expression [26,27,28]. Laboratory studies have confirmed the effect of tick-borne pathogens and other microorganisms present in the tick microbiome on metabolic processes occurring in the organism of these arthropods [29,30,31,32]. It has been evidenced that a decrease in the quantities of reserve substances, e.g., lipids, is associated with an increase in the activity and aggressiveness of hungry ticks. This increases the chance of finding a potential host and raises the potential risk of pathogen transmission [33,34]. Lipid substances present in tick bodies serve structural, signaling, and energetic functions. They are also utilized by tick-borne pathogens and have an impact on their growth and development, evasion of the immune response of the target host, and severity of symptoms of tick-borne diseases [35,36,37].

The aim of the present study was to determine for the first-time quantitative parameters that can affect ticks’ host-seeking behavior, i.e., fresh body mass, reduced body mass, water mass, lipid-free body mass, lipid mass, and lipid index, characterizing questing *I. ricinus* ticks collected from vegetation during the spring peak of their activity in eastern Poland.

## 2. Results

In total, 102 adult *I. ricinus* ticks (64 females and 38 males) were collected during field sampling. The highest number of ticks was collected in the last ten days of May (second sampling day), at a temperature of 22.1 °C and 57.57% relative humidity (Table 1).

**Figure 1:**
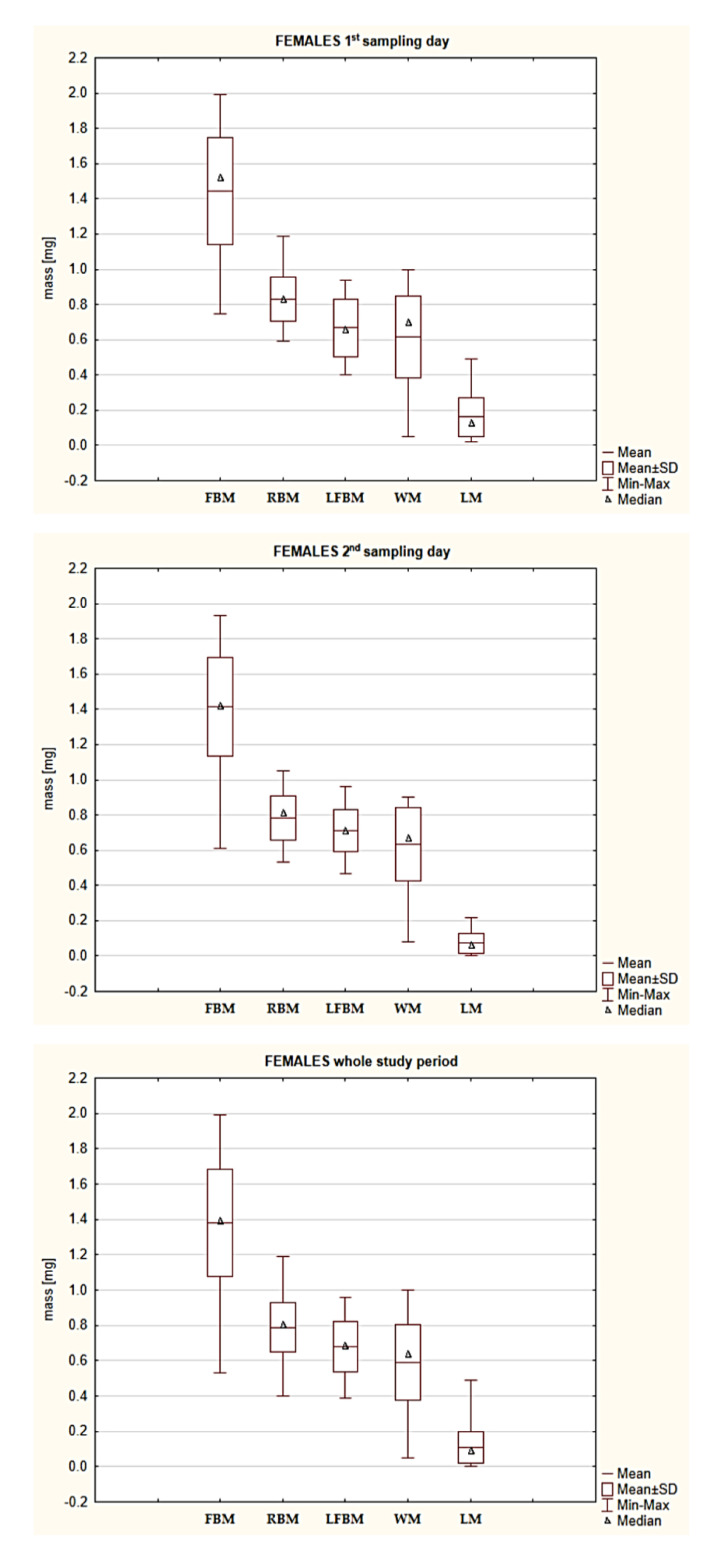
A quantitative comparison of the body components of *Ixodes ricinus* females. Box-plot diagram without outliers: Min-minimum; Max-maximum; SD-standard deviation; FBM-fresh body mass; RBM-reduced body mass; LFBM-lipid-free body mass; WM-water mass; LM-lipids mass.

**Figure 2:**
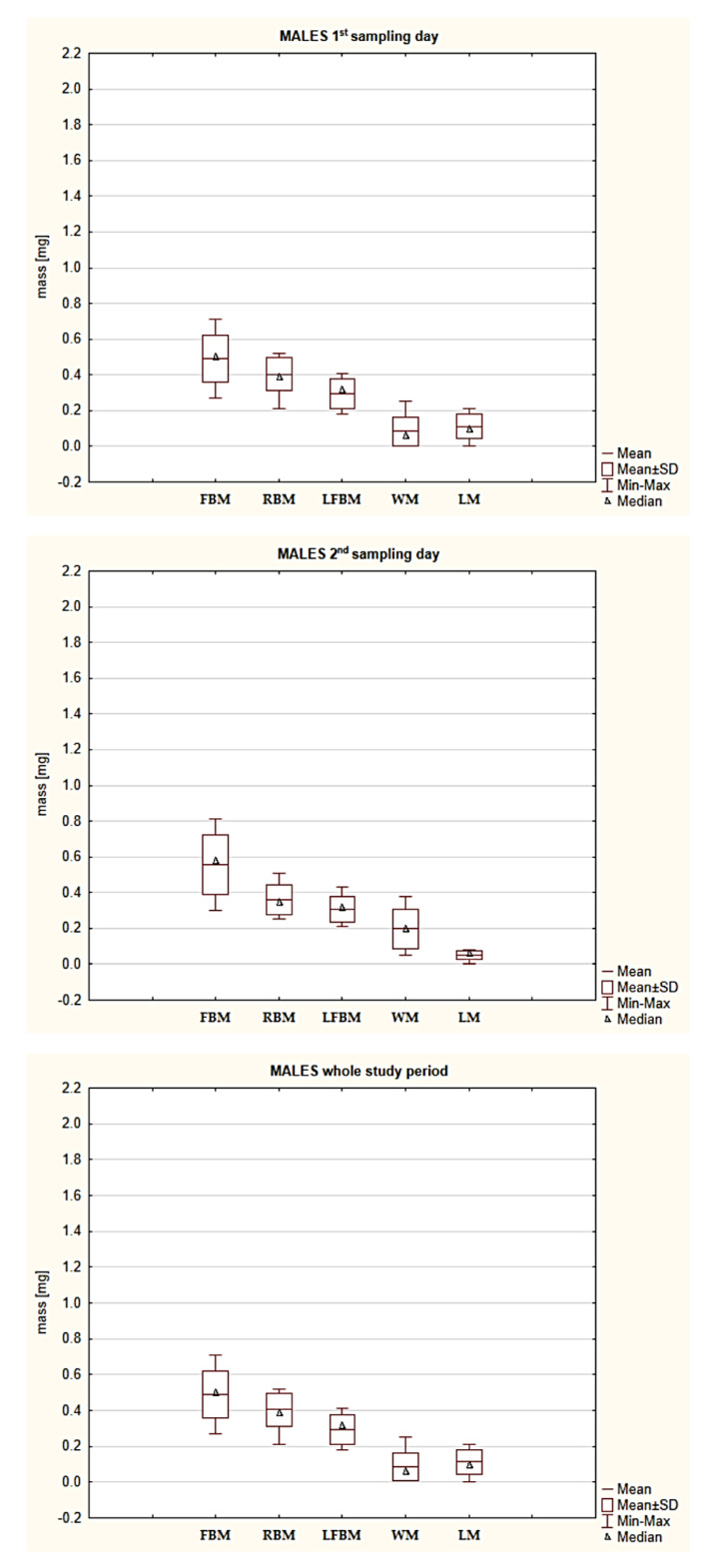
A quantitative comparison of the body components of collected *Ixodes ricinus* males. Box-plot diagram without outliers: Min-minimum; Max-maximum; SD-standard deviation; FBM-fresh body mass; RBM-reduced body mass; LFBM-lipid-free body mass; WM-water mass; LM-lipids mass.

The fresh body mass (FBM) ranged from 0.53 to 1.99 mg in the group of females and from 0.27 to 0.81 mg in the group of males (Table A1 and Table A2). The reduced body mass (RBM) of *I. ricinus* females and males ranged from 0.40 to 1.19 mg and from 0.21 to 0.52 mg, respectively (Table A1 and Table A2, Figure 1 and Figure 2). A statistically significant difference was found in the RBM between the groups of females collected on the first and second sampling days (Table 2, Figure 1). The water mass (WM) of the *I. ricinus* females and males ranged from 0.05 to 1.00 mg and from 0.01 to 0.38 mg, respectively (Table A1 and Table A2, Figure 1 and Figure 2). A statistically significant difference was found in the WM of males collected on the first and second sampling days (Table 2, Figure 2).

The lipid-free body mass (LFBM) of the *I. ricinus* females and males ranged from 0.39 to 0.96 mg and from 0.18 to 0.43 mg, respectively (Table A1 and Table A2, Figure 1 and Figure 2). A statistically significant difference was found in the LFBM of females and males (Table 2).

The lipid mass (LM) of the *I. ricinus* females and males reached values ranging from 0.00 to 0.49 mg and from 0.00 to 0.21 mg, respectively (Table A1 and Table A2, Figure 1 and Figure 2). There was a statistically significant difference in the mean LM between the *I. ricinus* females and males (Table 2) and in the LM values between females collected on the first and second sampling days (Table 2). A statistically significant difference was also found in the LM values of males collected on both sampling days (Table 2).

**Figure 3:**
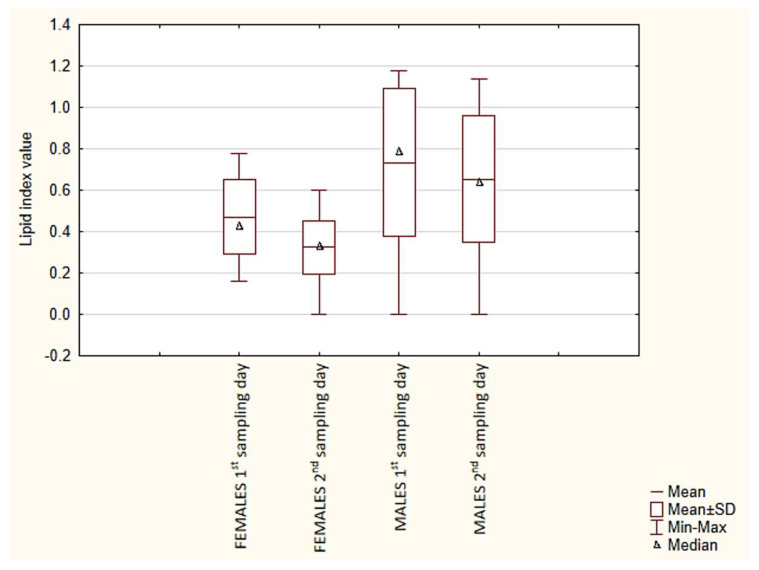
A comparison of the lipid index (LI) values in *Ixodes ricinus* females and males collected on the 1st and 2nd sampling days. A box-plot diagram without outliers: Min-minimum; Max-maximum; SD-standard deviation.

There was a moderate positive correlation between the RBM and LM of the *I. ricinus* females and males (Table 3, Table A1 and Table A2). Therefore, the comparison of the lipid content in the collected specimens was based on the lipid index (LI) value calculated with the formula proposed by Randolph et al. [38] and used by Burtis et al. [39]. The LI values of the *I. ricinus* females and males reached 0.78 and 1.18, respectively (Table A1 and Table A2, Figure 3).

A statistically significant difference was found in the LI values between females and males, in favor of males (Table 2). A statistically significant difference in the LI values between females and males from first sampling day was recorded (Table 2). There was a statistically significant difference in the mean LI values between the sexes of the ticks (Table 2).

There were statistically significant differences in the LM values between females collected on the first sampling day and on the second sampling day (*t* = 3.9965; *p* = 0.0001), as in the case of the LI values between these groups (*t* = 3.7796; *p* = 0.0002). Females from these groups differed statistically significantly in the RBM values (*t* = 2.3067; *p* = 0.0244) (Table 2).

**Figure 4:**
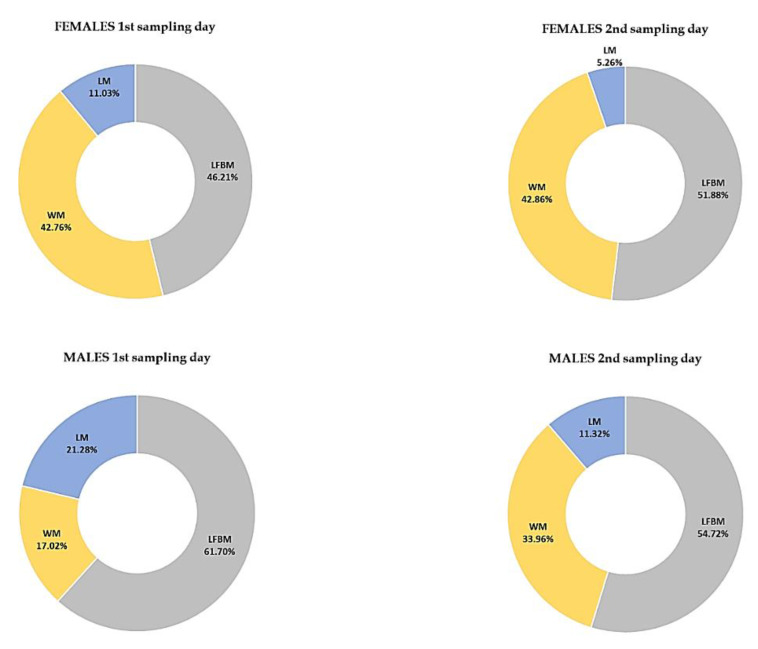
A comparison of the percentage of lipid-free body mass (LFBM), lipids mass (LM), and water mass (WM) fractions as fresh body mass (FBM) components in *Ixodes ricinus* ticks collected on the 1st and 2nd sampling days.

The statistically significant differences in the LM values between females and males and in the WM values between males collected on the first and second sampling days are reflected in the percentage contribution of these parameters to the body composition of the ticks (Figure 4).

**Figure 5:**
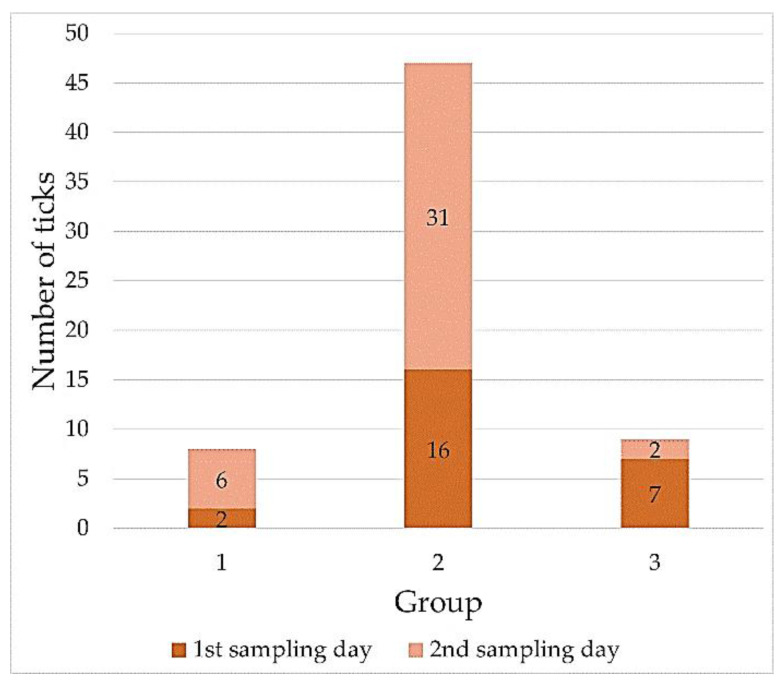
The number of *Ixodes ricinus* females in each range of the lipid index value.

**Figure 6:**
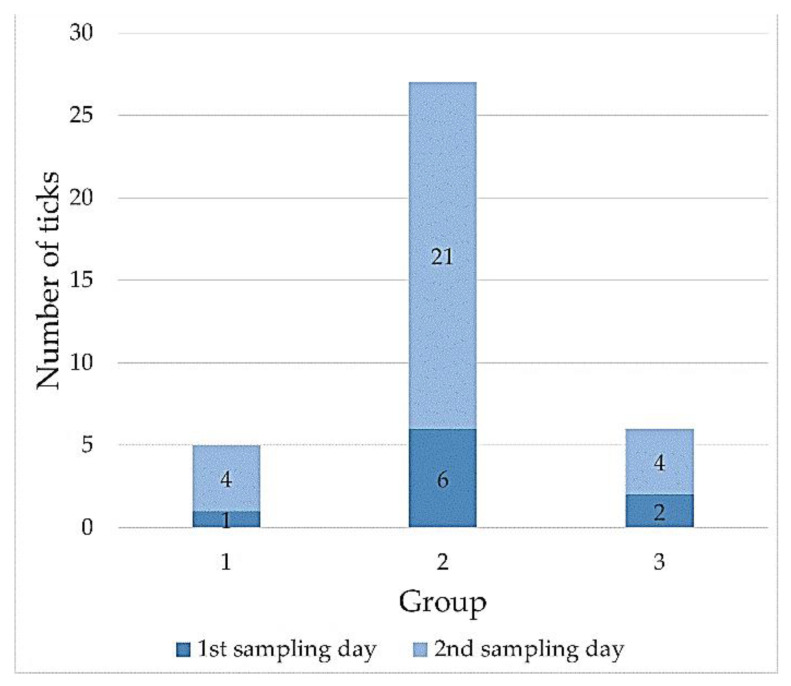
The number of *Ixodes ricinus* males in each range of the lipid index value.

The majority of the *I. ricinus* specimens collected on the first and second sampling days had LI values ranging from 0.2166 to 0.5463 (females) and from 0.3569 to 0.9884 (males) (Table 3, Table A1 and Table A2, Figure 5 and Figure 6).

## 3. Discussion

The authors are fully aware of the limitation of the study associated with the sample size. Nevertheless, the collection of *I. ricinus* ticks carried out at their activity peak should provide a group of specimens representing an optimal condition of the population. The values of one of the parameters (mean FBM ± SD) characterizing the questing *I. ricinus* ticks analyzed in this study were 1.38 ± 0.30 mg (females) and 0.53 ± 0.14 mg (males) (Table A1 and Table A2). Similar body weight of adult *I. ricinus* ticks was reported in other studies in Poland [40] and south-western England [41]. In our study, the values of FBM, RBM, WM, and LM did not differ statistically significantly between the *I. ricinus* females and males (Table A1 and Table A2). The normal distribution of the values of these parameters indicates that the active ticks are part of a homogeneous population. Therefore, it can be assumed that the conditions of the microhabitat in which the specimens were collected are optimal for these ticks.

The females and males collected on the first and second sampling days differed statistically significantly in the LM value (Table 2, Table A1 and Table A2). Besides quantitative changes in lipids occurring during the off-host phase [42,43], qualitative changes should be expected, i.e., seasonal changes in the fatty acid profile in adult *I. ricinus* specimens [44] and in the ratio of phospholipids to neutral lipids in active nymphs and adults [45]. Qualitative and quantitative changes in the concentration of lipid, glycogen, and protein metabolites are also a response to stressors affecting specimens (e.g., temperature). The rate of these changes varies largely depending on the developmental stage, with the highest values observed in *I. ricinus* females [45]. Differences in the utilization rate and qualitative composition of metabolites are indirectly indicated in our study, as evidenced by the statistically significant difference in the LI value (reducing the effect of the positive correlation between RBM and LM) in favor of males (Table 2, Table A1 and Table A2, Figure 3). The present findings agree with the results reported by Alasmari and Wall [45], showing a lower lipid utilization rate in tick males than in females. The rate of metabolism and consumption of reserve materials in ticks is also influenced by the presence of pathogens [29,30]. Noteworthy, LFBM is the most “stable” parameter of those analyzed in the present study, as there was no statistically significant difference within both sex groups between the ticks from the first and second sampling days. In turn, the value of LFBM differed statistically significantly between the females and males, which is a consequence of sexual dimorphism reflected by the substantially greater body mass of females (Table 2, Table A1 and Table A2, Figure 1 and Figure 2). The stability of the LFBM parameter (Table A1 and Table A2, Figure 4) is probably related to the fact that it no longer comprises lipid substances or water, but probably other chloroform-insoluble organic substances as well as anatomical structures and substances that are not decomposed or depleted during the starvation period, e.g., chitin [46].

The results of the present study indicate that the adult *I. ricinus* females and males collected on the second sampling day had statistically significantly lower mean LI values than the same-sex individuals collected on the first sampling day (Table 2, Table A1 and Table A2, Figure 3). This may suggest that they utilized greater amounts of lipids, as their activity and starvation periods were probably longer. An analysis of the activity of *I. ricinus* specimens of both sexes should take into account the saturation deficit effect. The mean 7-day saturation deficit values on the days preceding the tick collection and on the second sampling day were higher than the mean 7-day value during the days before and on the first sampling day. Nevertheless, the activity of the adult *I. ricinus* specimens collected on the second sampling day was higher than on the first sampling day (Table 1). The saturation deficit integrating temperature and relative humidity is an abiotic factor influencing the activity of ticks in the natural environment [42,47,48]. By enhancement of water loss through the body surface and increased transpiration through an open atrial valve in spiracles, saturation deficit forces ticks to move into lower layers of vegetation or even into the soil, where water is absorbed through the cuticle surface and via active water uptake, which interrupts the host-seeking activity [49,50,51,52]. Hence, we believe that our results may also support the hypothesis proposed by Uspensky [53] that low relative humidity and high air temperature increase aggressiveness in ixodid ticks, thereby creating more opportunities to find a host and allowing ticks to use the host’ blood as a water source for the rehydration process. The statistically significant difference in WM between the groups of males collected on the first and second sampling days (with higher values in the latter group) (Table 2, Figure 2 and Figure 4) is, contrary to what may seem, probably a consequence of the higher saturation deficit recorded during the 7 days preceding the second sampling. The males probably began to employ the aforementioned mechanisms of more intensive water absorption more effectively. Their activity on the first and second sampling days was lower (Table 1) in comparison with the group of females, where no statistically significant difference in WM was found between the two tick collection events (Table A1 and Table A2). Therefore, it can be assumed that some male specimens replenished water at that time; hence, they were not collected from the vegetation with the flagging method. Nevertheless, due to their biology, females are determined to seek a host and, therefore, their activity may be higher even in unfavorable conditions. Although the saturation deficit in the present study reached higher values than those reported by other researchers [54,55], the ticks were questing actively in the analyzed area (Table A1 and Table A2). This may also indicate a significant effect of other factors, e.g., the type of the microhabitat (with its vegetation cover) or the presence of potential hosts, which modify tick behavior and phenology via interactions at the local level [56,57].

The prolonged period of starvation in hard ticks also induces changes in the gene expression processes, thereby increasing tick activity and intensifying the host-seeking process [34], which may explain the higher activity of the females on the second sampling day with the lower mean LI value and the higher saturation deficit (Table 1, Table A1 and Table A2, Figure 3). Starving ticks are determined to find a host before the amounts of reserve substances drop to a critical level. Therefore, even in unfavorable environmental conditions (the high saturation deficit in the present study), ticks undertake questing in the “now or never” mode. A decrease in tick body weight during the starvation period, mainly caused by depletion of reserve materials, exerts an impact on the tonic immobility behavior. The several-month-long starvation period reduces the duration and frequency of employment of the tonic immobility mechanism (thanatosis) in *Dermacentor variabilis* and *Rhipicephalus sanguineus*, which can be regarded as an extension of the host-seeking time and enhancement of aggressiveness towards their natural enemies, which are then perceived as potential hosts [33]. A prolonged off-host phase has an effect on the parasitic phase, as it extends the feeding period, increases the amount of blood ingested by ticks, and increases the number of eggs in the batch, as shown in *D. variabilis* [58].

Ticks utilize energy from reserve materials accumulated mainly in the fat bodies and in the midgut [59]. In addition to the storage/energy function, fat bodies are involved in vitellogenesis in females and the production of antimicrobial peptides and proteins with various functions [60]. The rate of utilization of lipid materials varies depending on the development stage and external factors, and their depletion leads to tick death. As demonstrated by Steele and Randolph [61], *I. ricinus* nymphs utilize 0.00018 mg of lipids per day. The progressive decline in the mass of fatty bodies in ticks is regarded as an indicator of the condition and age of tick specimens and the age structure in tick populations. Researchers distinguish between the calendar age of ticks (describing chronologically the life of ticks from larval hatching to the death of an adult specimen or regarded as the duration of the developmental stages) and the biological/physiological age reflected in progressive consumption of nutrients and accumulation of metabolites [59,62,63]. The knowledge of the age structure of a vector population in a given ecosystem and determinants of the population dynamics facilitates assessment of the risk of tick attacks and pathogen transmission [64]. For the determination of the biological age and condition of hungry specimens, researchers use microscopic, anatomical, and histological methods [59,63,65,66,67], as well as quantitative and qualitative approaches [39,41,44,61] with the division of the pool of specimens into three, four, or even eight groups. The chloroform lipid extraction method used in the present study is believed to have higher accuracy and to reflect the changes taking place during tick aging better than morphometric methods [68].

The quantification of the lipid content used in the present study allowed the division of the ticks based on their LI values into three separate groups among the females and males (Table 3). On both the first and second sampling days, group 2 comprised the highest number of females and males, accounting for 73% and 75% of the collected ticks, respectively (Figure 5 and Figure 6). It can therefore be concluded that they represent the major age cohorts among the tick specimens. In turn, females and males with the highest LI values classified into group 3 (LI ∈ (0.5463; 0.7842〉 and LI ∈ (0.9884; 1.1765〉, respectively) (Table 3), which were collected on the second sampling day, are probably part of a cohort with a shorter activity period. They appeared in the population later, which may have been related to the delayed molting of nymphs feeding in late autumn [38]. The small proportion of physiologically young adults was also confirmed during the spring activity peak in a study conducted by Walker [69]. Another possible cause of such a high LI value in the specimens from group 3 in the present study is the probable infection by pathogens and their influence on lipid metabolism. The levels of lipids in *Borrelia* spp.-infected *I. ricinus* nymphs studied by Hermann et al. [70] were 12.1% higher than in non-infected nymphs. Noteworthy, the tick collection area in our study is a region with a confirmed prevalence of such pathogens as *Borrelia burgdorferi* s.l., *Anaplasma phagocytophilum*, *Babesia microti*, and TBE virus in *I. ricinus* ticks [71,72,73].

Since the Polesie National Park is an endemic area of tick-borne diseases [74,75,76], it is important to determine the population structure of the most important vector and one of the reservoirs of pathogens. This will allow estimation of the risk of tick attacks on humans and animals and selection of appropriate methods for the prevention of the transmission of this group of zoonotic diseases.

## 4. Materials and Methods

### 4.1. Study Area

**Figure 7:**
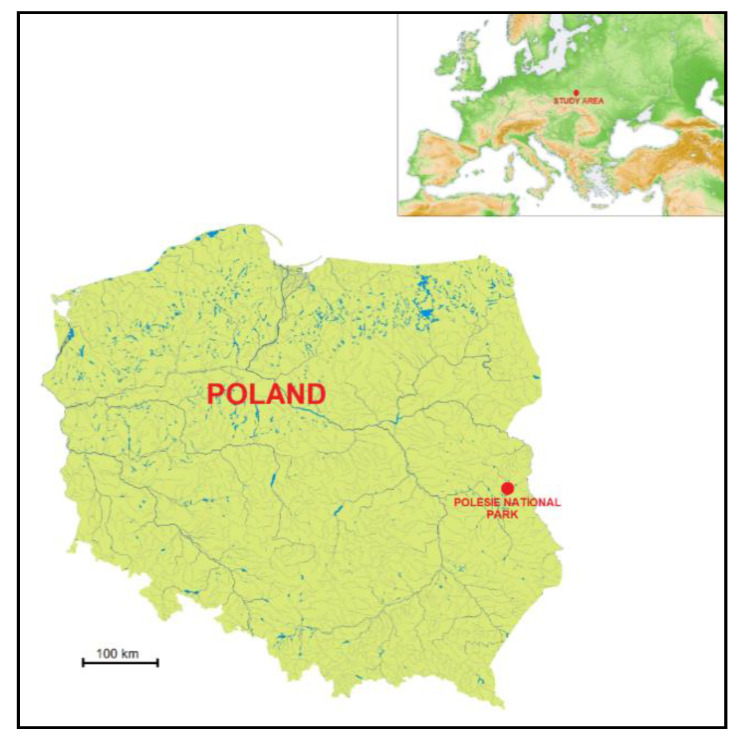
The localization of the study area; based on Wikipedia [77] and Wikimedia [78] with modifications.

The ticks were collected in the Polesie National Park located in central-eastern Poland (Figure 7). This approximately 100-km^2^ protected area with a minimal anthropogenic impact [74] is located in a region of endemic occurrence of tick-borne encephalitis and borreliosis [75,76].

The area of the Park lies in the temperate climate zone, with an average annual temperature of 7.4 °C and an annual precipitation sum of 542 mm [74].

The ticks were collected along transects overgrown by Tilio-Carpinetum oak-hornbeam vegetation and Ribeso nigri-Alnetum alder carr. These habitats are preferred by rodents from the genera *Apodemus*, *Microtus*, *Myodes*, and other small and medium-sized mammals (*Sorex* spp., *Myotis* spp., *Pipistrellus* spp., *Martes* spp., *Mustela* spp., *Erinaceus roumanicus*), cervids (Cervidae), wild boar (*Sus scrofa*), foxes (*Vulpes vulpes*), raccoon dogs (*Nyctereutes procyonoides)*, and wolves (*Canis lupus*). Many species of birds and reptiles live in the Park as well [79]. These species may be potential hosts of juvenile and adult stages of *Ixodes* ticks [9,80,81,82,83,84,85].

### 4.2. Tick Surveillance

*I. ricinus* ticks were collected on the first (1st sampling day) and last ten days (2nd sampling day) of May 2018. It is a period of the spring peak activity of adults of this species, dominant in the area, which is associated with the highest risk of infestations by these ticks [40,86,87]. The number of the other collected development stages (larvae and nymphs) did not ensure the reliability of the analysis; therefore, they were excluded from the study.

The flagging method was used for tick collection, following the procedure described by Nowak-Chmura [8]. The vegetation was swept with a 1-m^2^ flannel cloth, and the fabric was inspected after sweeping over every 2 m. The attached ticks were transferred with tweezers to a 100-cm^3^ plastic container containing a moist cotton swab to provide approx. 75% relative humidity. Immediately after collection, the ticks were transported to the laboratory and placed in a low-temperature freezer (manufactured by Arctico, Esbjerg, Denmark) at −80 °C to stop metabolism and prevent further water and lipid loss.

Next, the species, sex, and developmental stage of the ticks were identified using a Zeiss STEMI DV4 stereoscopic microscope (Carl Zeiss Light Microscopy, Göttingen, Germany) and a tick identification key [8].

### 4.3. Determination of the Parameters of I. ricinus Ticks

#### 4.3.1. Impact of Weather Conditions on *I. ricinus* Tick Activity in the Environment

A formula for the calculation of the saturation deficit was used to determine the impact of the weather conditions on the activity of the ticks in the habitat [40]. The saturation deficit value was calculated for the sampling days and 7-day periods prior to the sampling (averaged value) [88].

$$\mathrm{StDf}=\left(1-\frac{\mathrm{RH}}{100}\right)\times 4.9463\times e^{0.0621\times T}\;$$

where StDf is the saturation deficit; RH is the relative humidity; e is the actual vapor pressure; T is the temperature.

The weather data for the study area, i.e., temperature and relative humidity, were provided by tutiempo.net for the Włodawa weather station [89].

#### 4.3.2. Determination of the Values of Fresh Body Mass, Reduced Body Mass, Water Mass, Lipid-free Body Mass, Lipid Mass, and Lipid Index

The quantitative lipid determination method described by Steele and Randolph [59] was used to determine the values of parameters characterizing the collected tick specimens. This method consists of several successive stages: weighing individual ticks, drying (at 70 °C for 24 h), re-weighing, three-fold immersion in chloroform (each time for 24 h), subsequent drying, and weighing. Each *I. ricinus* specimen was weighed individually using an AS.R2 Plus analytical balance (RADWAG, Radom, Poland) with an accuracy of 0.01 mg.

This multi-stage method yielded values of parameters characterizing each tick, i.e., fresh body mass (FBM), reduced body mass (RBM), and lipid-free body mass (LFBM). The results obtained in this way were used for the calculation of the following parameters:

- water mass:
  WM=FBM−RBM
  where WM is the water mass; FBM is the fresh body mass; RBM is the reduced body mass
- lipids mass:
  LM=RBM−LFBM
  where LM is the lipids mass; RBM is the reduced body mass; LFBM is the lipid-free body mass
- lipid index value:
  Given the probability of a relationship between lipid mass and body mass, the lipid index was calculated for each specimen with a formula used by Randolph et al. [68]:
  $$\mathrm{LI}=\frac{\sqrt{\mathrm{LM}}}{\mathrm{RBM}}$$
  where LI is the lipid index; LM is the lipids mass; RBM is the reduced body mass.

#### 4.3.3. Classification of *Ixodes ricinus* Based on the Lipid Index Values

Based on the LI values, three groups were distinguished separately for the males and females, and the ticks were assigned as follows:

Group 1: LI ∈〈Min, Mean−SD)
Group 2: LI ∈〈Mean−SD, Mean+SD〉
Group 3: LI ∈(Mean+SD, Max〉

where: LI—lipid index; ∈—belongs to; Min—minimal lipid index value; Max—maximal lipid index value; Mean—mean lipid index value; SD—standard deviation.

### 4.4. Statistical Analysis

Since the data had a normal distribution, confirmed by the Kolmogorov-Smirnov test of normality, parametric tests were used to evaluate the statistical differences between the groups of ticks.

The statistical significance of differences between the mean weight of the lipids in the female and male ticks was determined using the T-Test for 2 Independent Means. The Single Sample T-Test was used to test the significance of differences in the FBM, RBM, LFBM, WM, and LI values within each group. In turn, the correlation between LM and RBM was checked with the Pearson correlation coefficient. The value of *p* < 0.05 was considered statistically significant. Statistical calculations were performed using the STATISTICA 10 PL statistical package (StatSoft, TIBCO Software Inc, Palo Alto, CA, USA).

For investigated qualitative parameters minimum, maximum, mean and standard deviation values were calculated.

## 5. Conclusions

At the beginning of the spring peak of *I. ricinus* activity in eastern Poland, specimens with higher lipid content and a higher lipid index dominated. Ticks with low lipid content, constituting the physiologically oldest group, were found to undertake host-seeking activity even in unfavorable humidity conditions, thus risking excessive water loss.

## Figures and Tables

**Table 1:** Weather parameters in the study area and the number of collected *Ixodes ricinus* ticks.

Sampling	T [°C]	RH [%]	StDf [mmHg]	Number of Active Ticks	
				F	M
1st sampling day	18.10	54.00	20.47	25	9
Mean for 7 previous days	17.47	56.43	22.10	-	-
2nd sampling day	22.1	48.00	57.25	39	29
Mean for 7 previous days	19.50	57.57	32.47	-	-

T—temperature; RH—relative humidity; StDf—saturation deficit; F—females; M—males.

**Table 2:** The results of statistical analysis.

	*t* Value	*p* Value	*r* Value
RBM of females collected on the 1st and 2nd sampling days	2.3067	0.0244	-
WM of males collected on the 1st and 2nd sampling days	−2.5079	0.0168	-
LFBM of females and males	15.6765	<0.0001	-
Mean LM between females and males	−2.1839	0.0157	-
LM between females collected on the 1st and 2nd sampling days	3.9965	0.0001	-
LM of males collected on the 1st and the 2nd sampling days	2.8045	0.0040	-
Correlation between the RBM and LM of females	-	0.0112	*r*_(62)_ = 0.3153
Correlation between the RBM and LM of males	-	0.0245	*r*_(36)_ = 0.3644
LI values between females and males	−6.1192	<0.0001	-
LI values between females collected on the 1st and the 2nd sampling days	3.7796	0.0002	-
LI of females and males collected on 1st sampling day	−2.8502	0.0038	-

RBM—reduced body mass; WM—water mass; LFBM—lipid-free body mass; LM—lipids mass; LI—lipid index; the value of *p* < 0.05 was considered statistically significant.

**Table 3:** Ranges of lipid index (LI) values in each group of *Ixodes ricinus* specimens.

	Group 1	Group 2	Group 3
F	LI ∈〈0.0000, 0.2166)	LI ∈〈0.2166, 0.5463〉	LI ∈(0.5463, 0.7842〉
M	LI ∈〈0.0000, 0.3569)	LI ∈〈0.3569, 0.9884〉	LI ∈(0.3569, 1.1765〉

LI—lipid index; F—females; M—males; ∈–belongs to.

## Appendix A

**Table A1:** Parameters of *Ixodes ricinus* females collected on the 1st and 2nd sampling days.

	F	FBM [mg]	RBM [mg]	LFBM [mg]	WM [mg]	LM [mg]	LI
1st sampling day	1	1.29	0.83	0.77	0.46	0.06	0.30
	2	1.86	0.94	0.91	0.92	0.03	0.18
	3	1.18	0.73	0.60	0.45	0.13	0.49
	4	1.27	0.59	0.49	0.68	0.10	0.54
	5	1.99	1.19	0.94	0.80	0.25	0.42
	6	1.49	0.95	0.76	0.54	0.19	0.46
	7	1.08	0.77	0.44	0.31	0.33	0.75
	8	1.36	0.73	0.63	0.63	0.10	0.43
	9	1.29	0.70	0.43	0.59	0.27	0.74
	10	1.55	0.80	0.66	0.75	0.14	0.47
	11	1.65	0.92	0.78	0.73	0.14	0.41
	12	1.52	0.87	0.78	0.65	0.09	0.34
	13	1.56	0.82	0.60	0.74	0.22	0.57
	14	1.53	0.82	0.64	0.71	0.18	0.52
	15	1.71	0.93	0.82	0.78	0.11	0.36
	16	1.49	0.77	0.70	0.72	0.07	0.34
	17	1.87	0.87	0.85	1.00	0.02	0.16
	18	1.67	0.93	0.66	0.74	0.27	0.56
	19	1.66	0.91	0.83	0.75	0.08	0.31
	20	0.96	0.91	0.42	0.05	0.49	0.77
	21	1.53	0.83	0.70	0.70	0.13	0.43
	22	1.59	0.84	0.80	0.75	0.04	0.24
	23	1.38	0.77	0.66	0.61	0.11	0.43
	24	0.92	0.71	0.40	0.21	0.31	0.78
	25	0.75	0.59	0.40	0.16	0.19	0.74
2nd sampling day	26	1.54	0.92	0.81	0.62	0.11	0.36
	27	1.34	0.70	0.66	0.64	0.04	0.29
	28	1.57	0.96	0.88	0.61	0.08	0.29
	29	0.61	0.53	0.47	0.08	0.06	0.46
	30	1.40	0.73	0.71	0.67	0.02	0.19
	31	1.69	0.81	0.74	0.88	0.07	0.33
	32	0.96	0.87	0.77	0.09	0.10	0.36
	33	1.60	0.90	0.82	0.70	0.08	0.31
	34	1.24	0.69	0.66	0.55	0.03	0.25
	35	1.56	0.83	0.65	0.73	0.18	0.51
	36	1.82	0.96	0.88	0.86	0.08	0.29
	37	1.12	0.70	0.69	0.42	0.01	0.14
	38	1.21	0.60	0.54	0.61	0.06	0.41
	39	1.18	0.63	0.61	0.55	0.02	0.22
	40	1.54	0.87	0.83	0.67	0.04	0.23
	41	1.23	0.83	0.61	0.40	0.22	0.57
	42	1.93	1.05	0.96	0.88	0.09	0.29
	43	1.30	0.65	0.65	0.65	0.00 *	0.00
	44	1.63	0.86	0.75	0.77	0.11	0.39
	45	1.42	0.68	0.65	0.74	0.03	0.25
	46	1.70	0.80	0.75	0.90	0.05	0.28
	47	1.57	0.83	0.81	0.74	0.02	0.17
	48	1.39	0.69	0.52	0.70	0.17	0.60
	49	1.41	0.68	0.66	0.73	0.02	0.21
	50	1.44	0.81	0.72	0.63	0.09	0.37
	51	0.96	0.58	0.53	0.38	0.05	0.39
	52	1.29	0.77	0.68	0.52	0.09	0.39
	53	1.21	0.78	0.63	0.43	0.15	0.50
	54	1.33	0.80	0.70	0.53	0.10	0.40
	55	0.89	0.45	0.45	0.44	0.00 *	0.00
	56	1.29	0.81	0.74	0.48	0.07	0.33
	57	1.05	0.62	0.57	0.43	0.05	0.36
	58	1.42	0.78	0.70	0.64	0.08	0.36
	59	1.75	1.02	0.89	0.73	0.13	0.35
	60	1.16	0.72	0.62	0.44	0.10	0.44
	61	1.27	0.82	0.75	0.45	0.07	0.32
	62	1.38	0.70	0.63	0.68	0.07	0.38
	63	0.53	0.40	0.39	0.13	0.01	0.25
	64	1.23	0.91	0.76	0.32	0.15	0.43
1st sampling day	Mean ± SD	1.45 ± 0.30	0.83 ± 0.13	0.67 ± 0.16	0.62 ± 0.23	0.16 ± 0.11	0.47 ± 0.18
	Min	0.75	0.59	0.40	0.05	0.02	0.16
	Max	1.99	1.19	0.94	1.00	0.49	0.78
2nd sampling day	Mean ± SD	1.34 ± 0.30	0.76 ± 0.14	0.69 ± 0.13	0.57 ± 0.21	0.07 ± 0.05	0.32 ± 0.13
	Min	0.53	0.40	0.39	0.08	0.00 *	0.00
	Max	1.93	1.05	0.96	0.90	0.22	0.60
Total	Mean ± SD	1.38 ± 0.3	0.79 ± 0.14	0.68 ± 0.14	0.59 ± 0.21	0.11 ± 0.09	0.38 ± 0.16
	Min	0.53	0.40	0.39	0.05	0.00 *	0.00
	Max	1.99	1.19	0.96	1.00	0.49	0.78

* Value lower than 0.01 mg; F—females; FBM—fresh body mass; RBM—reduced body mass; WM—water mass; LFBM—lipid-free body mass; LM—lipids mass; LI—lipid index; Mean—mean value; SD—standard deviation.

**Table A2:** Parameters of *Ixodes ricinus* males collected on the 1st and 2nd sampling days.

		M	FBM [mg]	RBM [mg]	LFBM [mg]	WM [mg]	LM [mg]	LI
1st sampling day		1	0.50	0.46	0.41	0.04	0.05	0.49
		2	0.27	0.21	0.21	0.06	0.00 *	0.00
		3	0.36	0.34	0.18	0.02	0.16	1.18
		4	0.50	0.38	0.32	0.12	0.06	0.64
		5	0.55	0.39	0.29	0.16	0.10	0.81
		6	0.52	0.49	0.33	0.03	0.16	0.82
		7	0.71	0.46	0.36	0.25	0.10	0.69
		8	0.40	0.39	0.18	0.01	0.21	1.18
		9	0.59	0.52	0.35	0.07	0.17	0.79
2nd sampling day		10	0.42	0.27	0.21	0.15	0.06	0.91
		11	0.81	0.51	0.43	0.30	0.08	0.55
		12	0.61	0.34	0.27	0.27	0.07	0.78
		13	0.58	0.38	0.33	0.20	0.05	0.59
		14	0.73	0.35	0.33	0.38	0.02	0.40
		15	0.65	0.44	0.39	0.21	0.05	0.51
		16	0.37	0.32	0.25	0.05	0.07	0.83
		17	0.55	0.38	0.32	0.17	0.06	0.64
		18	0.30	0.25	0.25	0.05	0.00 *	0.00
		19	0.61	0.29	0.18	0.32	0.11	1.14
		20	0.34	0.28	0.19	0.06	0.09	1.07
		21	0.68	0.44	0.40	0.24	0.04	0.45
		22	0.72	0.37	0.30	0.35	0.07	0.72
		23	0.44	0.42	0.36	0.02	0.06	0.58
		24	0.58	0.29	0.26	0.29	0.03	0.60
		25	0.73	0.45	0.42	0.28	0.03	0.38
		26	0.52	0.41	0.25	0.11	0.16	0.98
		27	0.48	0.39	0.30	0.09	0.09	0.77
		28	0.68	0.44	0.37	0.24	0.07	0.60
		29	0.55	0.28	0.27	0.27	0.01	0.36
		30	0.52	0.38	0.37	0.14	0.01	0.26
		31	0.67	0.42	0.31	0.25	0.11	0.79
		32	0.35	0.30	0.22	0.05	0.08	0.94
		33	0.41	0.36	0.24	0.05	0.12	0.96
		34	0.57	0.35	0.34	0.22	0.01	0.29
		35	0.67	0.34	0.34	0.33	0.00 *	0.00
		36	0.44	0.27	0.19	0.17	0.08	1.05
		37	0.33	0.30	0.25	0.03	0.05	0.75
		38	0.36	0.31	0.20	0.05	0.11	1.07
1st sampling day		Mean ± SD	0.49 ± 0.12	0.40 ± 0.09	0.29 ± 0.08	0.08 ± 0.07	0.11 ± 0.06	0.73 ± 0.36
		Min	0.27	0.21	0.18	0.01	0.00 *	0.00
		Max	0.71	0.52	0.41	0.25	0.21	1.18
2nd sampling day		Mean ± SD	0.54 ± 0.14	0.36 ± 0.07	0.29 ± 0.07	0.18 ± 0.11	0.06 ± 0.04	0.65 ± 0.31
		Min	0.30	0.25	0.18	0.02	0.00 *	0.00
		Max	0.81	0.51	0.43	0.38	0.16	1.14
Total		Mean ± SD	0.53 ± 0.14	0.37 ± 0.08	0.29 ± 0.07	0.16 ± 0.11	0.07 ± 0.05	0.67 ± 0.32
		Min	0.27	0.21	0.18	0.01	0.00 *	0.00
		Max	0.81	0.52	0.43	0.38	0.21	1.18

* Value lower than 0.01 mg; M—males; FBM—fresh body mass; RBM—reduced body mass; WM—water mass; LFBM—lipid-free body mass; LM—lipids mass; LI—lipid index.
